# Supplementary material for: Contribution of oxic methane production to surface methane emission in lakes and its global importance
Source: Nat Commun. 2019 Dec 2;10:5497. doi: 10.1038/s41467-019-13320-0 (PMC6888895; doi:10.1038/s41467-019-13320-0)
Supplement: Supplementary file 1 — Supplementary Information [file 41467_2019_13320_MOESM1_ESM.pdf]

Supplementary Information for

Contribution of oxic methane production to surface methane emission in lakes and its global importance

by Günthel et al.

<u>Supplementary Note 1:</u> Re-analysis of Lake Hallwil surface mixed layer (SML) methane mass balance (June-August 2016)

We re-analyzed the Lake Hallwil methane mass balance[1] applying a bathymetry based on the Swiss topographic map of the lake, which was confirmed by a bathymetric survey[2]. The analysis was performed reconsidering the key variables for the methane budget: (a) littoral methane flux contribution and (b) methane evasion to the atmosphere. The other budget components (methane oxidation, input from rivers and diffusion from the hypolimnion) play a minor role in this system and were applied as previously described[1]:

Re-calculation of Lake Hallwil bathymetry. The bathymetry of Lake Hallwil used in Donis et al. (2017)[1] is the result of a seismic survey carried out in 2015[1]. These measurements lead to the estimation of the lake planar area and sediment area used for the methane mass balance of the surface mixed layer. When estimating these parameters based on a geometric extrapolation from the isolines of the topographic map of Lake Hallwil (https://www.swisstopo.admin.ch/en/home.html), the littoral area was underestimated by a factor 7 by Donis et al. (2017)[1]. Our map-based calculations give a surface lake area of 9.9 * 10^6 m^2 instead of 8.4 * 10^6 m^2 as previously reported in Donis et al. (2017)[1]. The sediment area of the surface mixed layer (5 m deep) is 0.7 * 10^6 m^2 instead of 0.1 * 10^6 m^2, and consequently the surface mixed layer volume equals 48 * 10^6 m^3 instead of 41 * 10^6 m^3.

Lake Hallwil littoral contribution to SML methane budget. As revealed from a survey of surface methane concentration carried out in September 2016, Lake Hallwil's littoral methane production is restricted to some areas of the lake sediments (Supplementary Fig. 1).

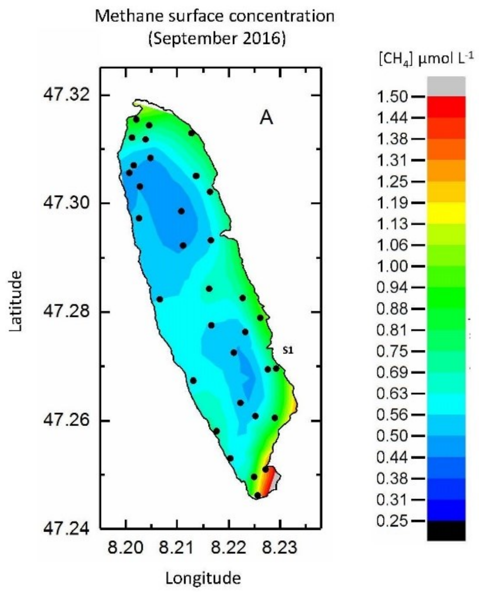

Supplementary Figure 1 l Methane surface water concentrations in Lake Hallwil (9th September 2016). Black dots represent the sampling points. S1 indicates the location of measured sediment fluxes - diffusive and ebullitive (see Methods in Donis et al. 2017[1]). Source data are provided as a Source Data file.

Sediment methane diffusive fluxes were measured in one of the lake hotspots in September 2016 (S1 in Supplementary Fig. 1 above and Supplementary Fig. 1 in Donis et al. 2017[1]). The average diffusive flux value of (mean±SD) 1.75±0.2 mmol m^{-2} d^{-1} was implemented in the SML budget and assumed to be constant from June to September for the entire littoral sediment area. Similarly, a littoral methane ebullition rate of (mean±SD) 1.2±0.8 mmol m^{-2} d^{-1}, as reported for the same site (S1) by Flury et al. 2010[3], was applied to the entire sediment area assuming that bubbles contained 100 % CH_4 that dissolved into the water column and homogeneously distributed in the surface mixed layer.

This conservative approach was adopted to compensate for the uncertainty intrinsic to a system-wide analysis based on discrete measurements. Applying the measured flux obtained from a hot spot to the entire littoral zone provides a conservative estimate of the littoral contribution to the methane concentration in the pelagic surface layer.

Lake Hallwil – surface methane emission. Donis et al.[1] determined the surface methane emission in Lake Hallwil in three ways: (a) in situ measurements with floating chambers, (b) k_{600}-calculations based on wind relationship (k_{600} = 2 * U_{10}), (c) k_{600}-calculations using wind relationships from MacIntyre et al. (2010)[4] for heated water columns (positive buoyancy flux, k_{600} = 1.74 * U_{10} - 0.15). We added 3 additional parametrizations: based on wind speed and lake size from Vachon and Prairie (2013)[5] (k_{600} = 2.51 + 1.48 * U_{10} + 0.39 * U_{10} * $\log_{10}$[lake area]), and for cooling and mixing water column from MacIntyre et al. (2010)[4] (negative buoyancy flux, k_{600} = 2.04 * U_{10} + 2, and all buoyancy fluxes combined, k_{600} = 2.25 * U_{10} + 0.16). We solved for the corresponding flux using Fick's First Law with an average surface CH_4 concentration of 0.3 µmol l^{-1} as reported in Donis et al. (2017)[1]. Results are summarized in Supplementary Table 1:

Supplementary Table 1 I Surface methane fluxes in Lake Hallwil.

Type of model	April – August [mmol m^{-2} d^{-1}]	June – August [mmol m^{-2} d^{-1}]
Flux chamber	0.6±0.3	0.8±0.1
Hallwil relationship	0.8±0.5	0.8±0.2
MacIntyre et al. (2010)		
- positive buoyancy flux	0.7±0.4	1.0±0.2
- negative buoyancy flux	1.3±0.5	1.6±0.5
- combined buoyancy fluxes	1.0±0.5	1.1±0.6
Vachon and Prairie (2013)	1.4±0.5	1.7±0.2

Listed values as mean±SD

Lake Hallwil SML methane mass balance. Lake Hallwil methane mass balance for the SML was recalculated (equation (3) in Donis et al. 2017[1]) with the corrected planar area (A_p), sediment area (A_s) and mixed layer volume ($\forall$). In Supplementary Table 2, the lower bound of the mass balance represents the most conservative approach, i.e. using chamber measurements for surface flux (April to August) and littoral sediment flux including CH_4

ebullitive input. The upper bound represents the surface fluxes obtained by wind relationship from the study site and reasonably assuming ebullition as a negligible contribution to the pelagic surface methane concentrations.

Supplementary Table 2 | Lower and upper bound values of SML methane mass balance in Lake Hallwil (June – August).

**Mass balance component**	**Symbol**	**Lower bound [mol d^{-1}]**	**Upper bound [mol d^{-1}]**
Surface emission	F_S	5969±2984	7958±1989
Methane oxidation	*MOx*	150±8	150±8
Littoral ebullition	$F_{L,eb}$	134±89	0
Littoral diffusion	$F_{L,sed}$	196±22	196±22
River input	F_R	0-207	0-207
Diffusion from thermocline	F_z	252±84	252±84
Internal (oxic) production	$P_{net,s}$	3738±3055	6629±2014
Oxic methane contribution	OMC	63 %	83 %

Note – symbols as in Dohnis et al.[1] Values given as mean±SD

Using these inputs and assumptions, we calculated OMP rates between 78±63 (lower) and 138±42 nmol l^{-1} d^{-1} (upper bound); this production rates correspond to a minimum of 63 % and a maximum of 83 % contribution to total emissions from Lake Hallwil to the atmosphere.

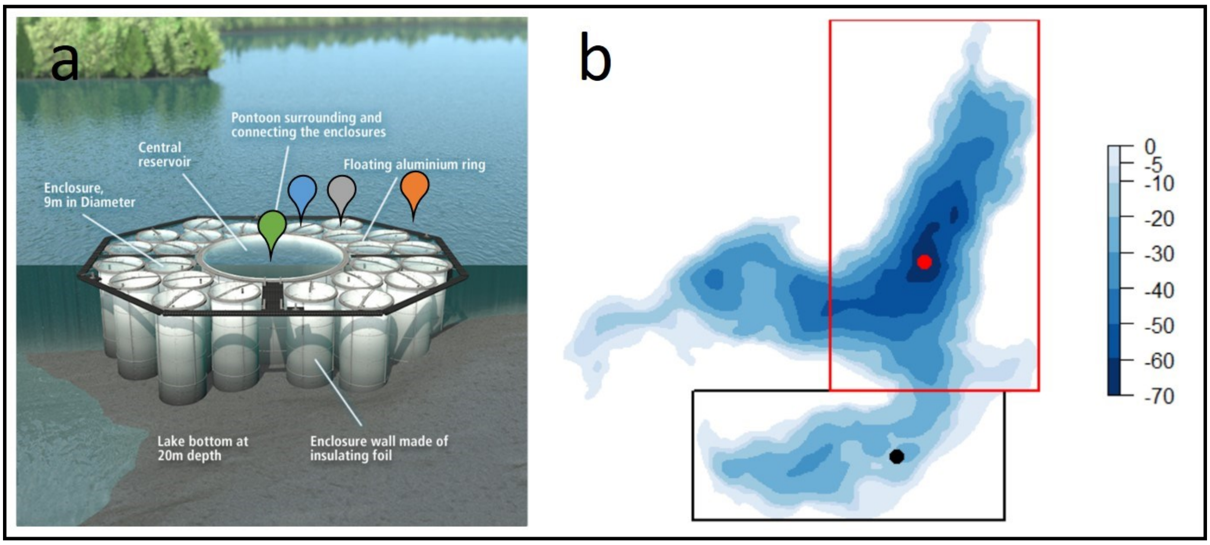

Supplementary Figure 2 | Illustration of the sampling sites and Lake Stechlin's bathymetry. Panel **(a)**, schematics of the lake lab facility (source: https://www.lake-lab.de/index.php/Design.html, picture modified) and related sampling locations: central reservoir (green), experimental enclosure 1 (blue), experimental enclosure 13 (grey) and the adjacent open water (orange). Panel **(b)** depicts the bathymetry of Lake Stechlin [m]. The lake has 3 basins: South (black frame), Northeast (red frame) and North-West basin. Seasonal methane measurements were done at the deepest point (69.5 m deep; red dot) and adjacent to the lake lab facility (20.5 m deep; black dot).

Supplementary Table 3 I Sampling schedule throughout 2014 – 2018. Detailed descriptions on how parameters were recorded can be found in the method section. Measurements in the experimental enclosures were taken 2 weeks after the water had been exchanged with lake water; in contrast, water in the central reservoir has never been exchanged since its installation in 2011/2012. Sampling locations are further described in Supplementary Figure 2.

Year	Month	Location	Purpose	n[a]	WC Profiles[b]	Surface emission[c]	Environmental parameters[d]
2014	Aug	exp. enclosure 1	quantify F_L	4 (SS)	yes	modelled	yes
2014	Aug	exp. enclosure 13	quantify F_L	5 (SS)	yes	modelled	yes
2014	Aug	South basin	quantify F_L	4 (SS)	yes	modelled	yes
2016	Mar-Jul	North basin	seasonal OMP,	6 (SS)	yes	measured (5/6)	yes
			basin variation	13 (NS)	yes	measured (13/13)	yes
2016	Mar-Jul	South basin	seasonal OMP,	6 (SS)	yes	modelled	yes
			basin variation	10 (NS)	yes	modelled	yes
2016	May, Jul	central reservoir	seasonal OMP,	1 (SS)	yes	modelled	yes
			isolated water	2 (NS)	yes	modelled	yes
2018	Jul	North basin	seasonal OMP, basin variation	1 (SS)	yes	measured (1/1)	yes
2018	Jul	South basin	seasonal OMP, basin variation	1 (SS)	yes	measured (1/1)	yes

(**a**) n represents the repetition of methane measurements (each taken on a different day during day time) including water column profile (water samples transferred into glass bottles, crimp-closed, He head space replacement, GC/FID analysis) and surface emission (floating chamber measurements), recordings were taken during the stratified season (SS) or non-stratified/intermediate season (NS); (**b**) WC profiles indicate water column methane profiles that were taken from the surface down to below the thermocline (ca. 5-7 m depth) in 1 to 2 m increments; (**c**) surface emission was measured (see methods) using a floating chamber or estimated from a wind based model developed from our own floating chamber measurements and compared to models in the literature (details in Supplementary Note 2); (**d**) environmental parameters include wind data that were recorded in 10 m above lake surface by the Neuglobsow weather station next to Lake Stechlin and were provided by the Umweltbundesamt, water temperature was recorded by automated YSI probes permanently mounted on the lake lab facility in the South basin (profiling the upper 20 m of the water column continuously in 60 min intervals); F_L is the lateral methane input and OMP is oxic methane production; in 2016 Lake Stechlin stratified ca. mid-May (see Supplementary Fig. 3a,b); samplings in 2014 and 2018 were done during stratification.

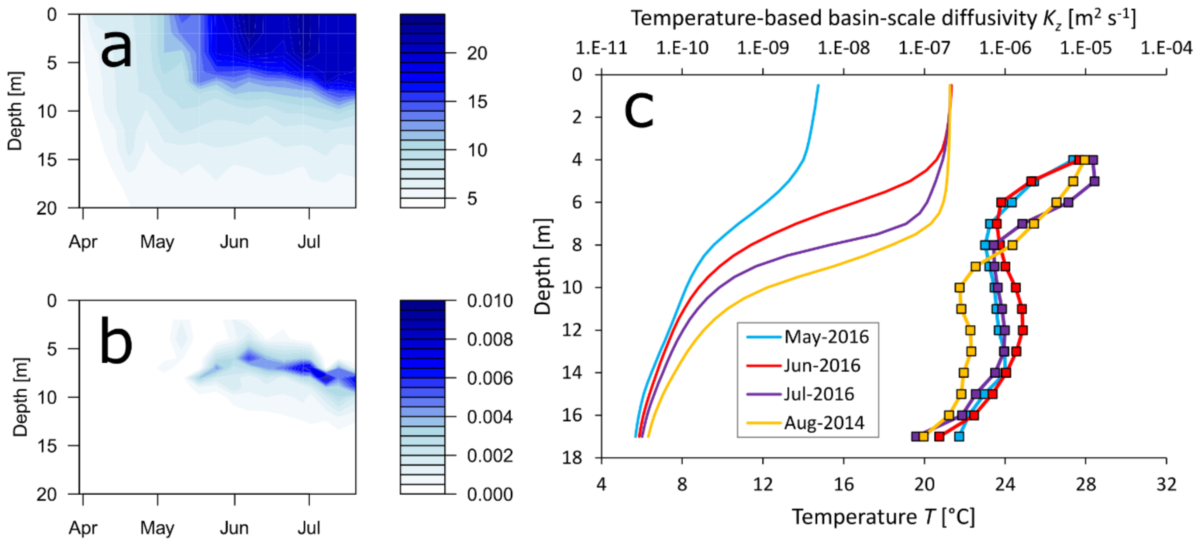

Supplementary Figure 3 | Physical characteristics of the water column. Panel **(a)**: Water column temperature [°C] in 2016 as recorded by a YSI probe. Panel **(b)**: Water column buoyancy frequency N^2 [s^{-1}] in 2016, computed from temperature profiles using the R-package rLakeAnalyzer[6]. Panel **(c)**: Water column temperature (T; smooth lines) recorded by automated YSI probes measuring continuously in a 30 min and 0.5 m interval and averaged monthly. Basin-scale diffusivity (K_z; squares) was calculated as after the heat-budget method from temperature data for stratified periods (monthly averages). Source data are provided as a Source Data file.

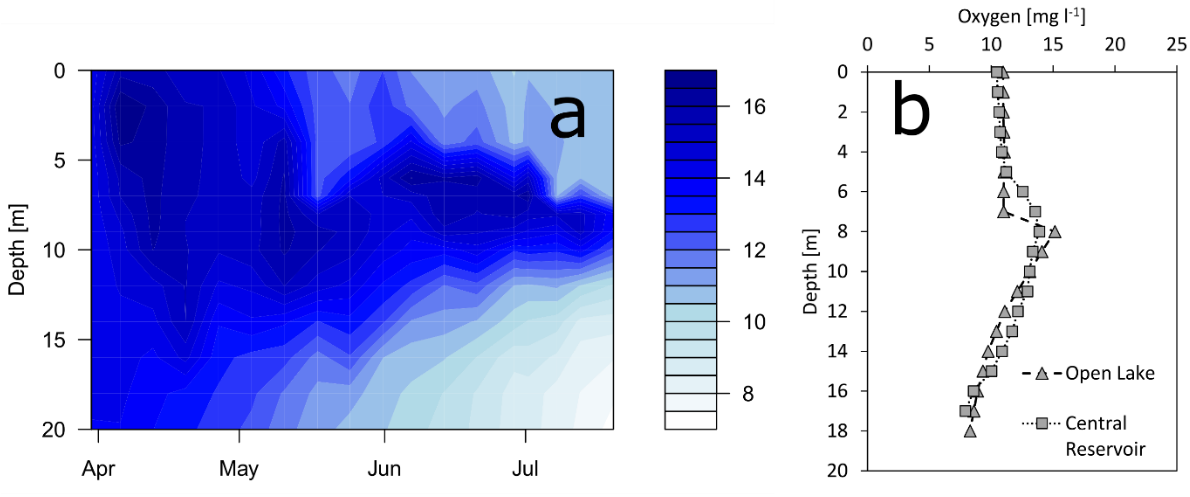

Supplementary Figure 4 | Oxygen measurements. Panel (**a**): Water column oxygen concentration [mg l^{-1}] in the open lake (South basin, 2016). Panel (**b**): Discrete measurements of water column oxygen concentration taken on 7th July 2016 in the open lake and inside the central reservoir. Oxygen was measured using a YSI probe. Source data are provided as a Source Data file.

Supplementary Note 2: Establishing the gas transfer constant (k_{600}) – wind speed (U_{10}) relationship

Using the general gas transfer formula (supplementary equation (S1)), the measured surface methane emission (F_S) and surface water methane (c_{water}), gas transfer constants (k_{CH4}) were calculated (atmospheric methane content c_{air} = 1.88 ppm) as:

$$k_{\mathrm{CH4}} = \frac{F_{\mathrm{S}}}{(c_{\mathrm{water}} - c_{\mathrm{air}})}; \text{ [cm h}^{-1}\text{]} \qquad \text{(S1)}$$

Subsequently, the k_{CH4} values were transformed into k_{600} values:

$$k_{600} = \frac{k_{\mathrm{CH4}}}{\left(\frac{Sc_{\mathrm{CH4}}}{600}\right)^{q}} \text{ ; [cm h}^{-1}\text{]} \qquad \text{(S2)}$$

Where Sc_{CH4} is the dimensionless Schmidt number (computed after Engle & Maleck[7]) and q is a conversion factor with a value of (-1/2) for wind speeds ≥3.7 m s^{-1} and (-2/3) for wind speeds below (Jähne et al.[8]). The k_{600} values were plotted over wind speed (U_{10}) and the obtained linear relationship was then used to estimate missing methane surface emissions. Supplementary Table 3 illustrates measured and estimated surface emissions and related parameters using the established gas-transfer model. The conversion of methane surface fluxes to k_{600} values and plotting over U_{10} yielded the following relationship: k_{600} [cm h^{-1}] = 1.98 * U_{10} [m s^{-1}] + 0.94 (R^2 = 0.44, p < 0.01) (Supplementary Table 4). Surface methane emission rates for the South basin were additionally computed using alternative gas-transfer models from the literature (Supplementary Table 7). Results indicate that our gas transfer model predicts values close to the mean values predicted by the alternative models.

Supplementary Table 4 | Data summary of parameters for estimating methane surface emission based on the k_{600}–wind relationship.

Date [dd/mm/yyyy]	Site	t [hh:mm]	U_{10} [m s^{-1}]	T_{SW} [°C]	$C_{CH4\ SW}$ [µmol l^{-1}]	L [mol l^{-1} atm^{-1}]	Sc [ ]	Δ_{CH4}[a] [µmol l^{-1}]	k_{CH4} [cm h^{-1}]	k_{600} [cm h^{-1}]	F_S [mmol m^{-2} d^{-1}]
08/03/2016	NE	14:00	3.9	3.2	0.02221	0.00235	1560.74	0.01779	7.012	11.309	0.030
15/03/2016	NE	14:00	2.3	3.3	0.03132	0.00234	1553.23	0.02691	5.000	9.427	0.032
21/03/2016	NE	12:00	4.7	3.7	0.04525	0.00232	1522.61	0.04089	8.743	13.927	0.086
30/03/2016	NE	10:00	3.1	4.2	0.04487	0.00228	1469.43	0.04059	4.749	8.629	0.046
05/04/2016	NE	13:30	1.3	7.3	0.06804	0.00209	1220.80	0.06410	3.246	5.212	0.050
12/04/2016	NE	12:30	1.4	6.7	0.07852	0.00213	1268.07	0.07452	2.015	3.319	0.036
26/04/2016	NE	12:00	3.1	8.1	0.13658	0.00205	1166.56	0.13272	6.578	10.248	0.210
03/05/2016	NE	13:00	1.9	9.7	0.11743	0.00197	1063.82	0.11373	3.175	4.651	0.087
10/05/2016	NE	12:00	3.8	13.5	0.18101	0.00179	858.09	0.17763	7.707	9.216	0.329
17/05/2016	NE	15:00	n. a.	13.0	0.19187	0.00182	884.33	0.18845	12.363	16.012	0.559
24/05/2016	NE	12:00	3.8	16.9	0.26583	0.00166	717.28	0.26271	5.152	5.633	0.325
31/05/2016	NE	12:00	0.6	19.1	0.35005	0.00159	644.75	0.34707	1.890	1.983	0.157
06/06/2016	NE	13:00	2.0	22.0	0.57289	0.00150	559.39	0.57008	3.545	3.383	0.485
13/06/2016	NE	13:00	1.4	19.5	0.43072	0.00157	632.03	0.42776	2.613	2.705	0.268
20/06/2016	NE	13:00	2.7	20.2	0.70184	0.00155	609.92	0.69892	6.247	6.299	1.048
07/07/2016	NE	14:30	3.4	20.1	0.36378	0.00155	613.14	0.36086	4.660	4.728	0.404
12/07/2016	NE	15:00	4.1	20.8	0.40794	0.00153	592.46	0.40506	3.493	3.471	0.340
19/07/2016	NE	15:00	2.5	20.0	0.38773	0.00156	614.61	0.38480	3.086	3.136	0.285
30/03/2016	S	12:30	3.3	4.7	0.04618	0.00225	1427.42	0.04195	4.855	7.489	0.049
05/04/2016	S	16:00	2.0	7.5	0.07498	0.00209	1212.91	0.07105	3.453	4.910	0.059
12/04/2016	S	15:00	1.2	7.6	0.11609	0.00208	1202.91	0.11218	2.347	3.323	0.063
19/04/2016	S	13:30	9.3	7.9	0.11907	0.00206	1183.86	0.11519	12.327	19.392	0.341
26/04/2016	S	14:30	2.0	8.6	0.13230	0.00203	1135.32	0.12849	3.569	4.910	0.110
03/05/2016	S	15:30	6.4	11.0	0.15123	0.00190	986.56	0.14765	9.790	13.639	0.347
10/05/2016	S	14:30	3.6	14.7	0.28610	0.00174	803.93	0.28282	6.984	8.084	0.474
17/05/2016	S	17:30	0.0	13.6	0.21174	0.00179	854.36	0.20838	0.790	0.942	0.039
24/05/2016	S	14:30	5.0	18.4	0.26537	0.00161	665.19	0.26235	10.140	10.861	0.638
31/05/2016	S	14:30	1.4	20.4	0.39583	0.00155	604.04	0.39293	3.707	3.720	0.350
06/06/2016	S	15:30	2.2	23.0	0.50423	0.00147	534.33	0.50147	5.623	5.307	0.677
13/06/2016	S	15:30	2.0	20.6	0.66523	0.00154	598.11	0.66234	4.918	4.910	0.782
20/06/2016	S	15:30	2.9	20.6	0.63610	0.00154	599.96	0.63320	6.696	6.695	1.018
07/07/2016	S	17:00	4.1	20.1	0.44668	0.00155	611.97	0.44376	8.957	9.076	0.954
12/07/2016	S	17:30	2.6	20.8	0.46059	0.00153	591.90	0.45771	6.142	6.100	0.675
19/07/2016	S	17:30	1.9	20.4	0.46982	0.00155	605.54	0.46691	4.690	4.712	0.526
03/05/2016	CR	16:30	6.7	11.0	0.01316	0.00190	985.72	0.00958	10.152	14.135	0.023
10/05/2016	CR	15:30	3.2	16.3	0.00707	0.00168	740.05	0.00391	6.475	7.191	0.006
07/07/2016	CR	18:00	2.8	20.1	0.01001	0.00155	612.85	0.00709	6.429	6.497	0.011
04-13/08/2014[b]	S	6-21:00	2.1	23.7	0.58419	0.00145	517.76	0.58146	5.506	5.115	0.768
04-13/08/2014[b]	E1	6-21:00	2.1	23.7	0.36739	0.00145	517.76	0.58146	5.506	5.115	0.482
04-13/08/2014[b]	E13	6-21:00	2.1	23.7	0.29207	0.00145	517.76	0.58146	5.506	5.115	0.382

Site – sampling site (Northeast basin – NE: 53°09'20.2"N 13°01'51.5"E / South basin – S: 53°08'35.8"N 13°01'43.2"E / central reservoir – CR: 53°08'35.8"N 13°01'41.1"E / enclosure 1 – E1: 53°08'36.4"N 13°01'41.6"E / enclosure 13 – E13: 53°08'36.5"N 13°01'42.1"E); t – time; U_{10} – wind speed recorded at 10 m above lake surface; T_{SW} – surface water temperature; $C_{CH4\ SW}$ – surface water methane concentration; L – methane solubility; Sc – Schmidt number; Δ_{CH4} – CH_4 gradient between surface water and air (= C_{water} – C_{air}); k – wind dependent gas transfer constants; F_S – methane water-to-air flux; [a] assuming 1.88 ppm methane content in the atmosphere; [b] corresponding parameters U_{10}, T_{SW} and $C_{CH4\ SW}$ were averaged for day times (06:00 – 21:00, local times) and given dates; n. a. – not available

Supplementary Table 5 | Mass balance components for estimating the lateral methane source. Oxic production rates computed for the mesocosm enclosures were applied to the mass balance for the open water giving the average lateral methane input.

Site	Mass Balance Component	Symbol	Whole System		Per Volume
			[mol d^{-1}]	[kg d^{-1}]	[nmol l^{-1} d^{-1}]
Experimental Enclosures	Surface emission	F_S	(2.7±0.5)E-2	(4.4±0.7)E-4	72.0±11.7
	Methane oxidation	*MOx*	1.2E-2	1.9E-4	30.3
	Lateral sediment input	F_L	0	0	0
	Diffusion from thermocline	F_z	(4.3±5.8)E-4	(6.9±9.3)E-6	1.1±1.5
	Internal (oxic) production	P_{net}	(3.9±0.6)E-2	(6.2±1.0)E-4	**101.1±16.8**
South basin	Surface emission	F_S	862.7	13.8	150.7
	Methane oxidation	*MOx*	173.7	2.8	30.3
	Lateral sediment input	F_L	437.1±67.2	7.0±1.1	**76.3±11.7**
	Diffusion from thermocline	F_z	19.1	0.3	3.3
	Internal (oxic) production	P_{net}	578.9±96.2	9.3±1.5	101.1±16.8

Measurements were taken inside experimental enclosure 1 (20 m deep; 53°08'36.4"N 13°01'41.6"E) and 13 (20 m deep; 53°08'36.5"N 13°01'42.1"E), as well as in the open water adjacent to the enclosures (20.5 m deep; 53°08'36.6"N 13°01'42.8"E) in the South basin of Lake Stechlin. Measurements were taken 4-5 times inside the enclosures and 4 times in the open lake on different days during the period 4-13th August 2014. Methane profiles and surface fluxes were averaged for the mass balance. Surface area: enclosures each 63.6 m^2, South basin 1,122,775 m^2. Monte Carlo simulation (9999 iterations) was used to solve the mass balance after the target component (in bold). Values listed are mean±SD.

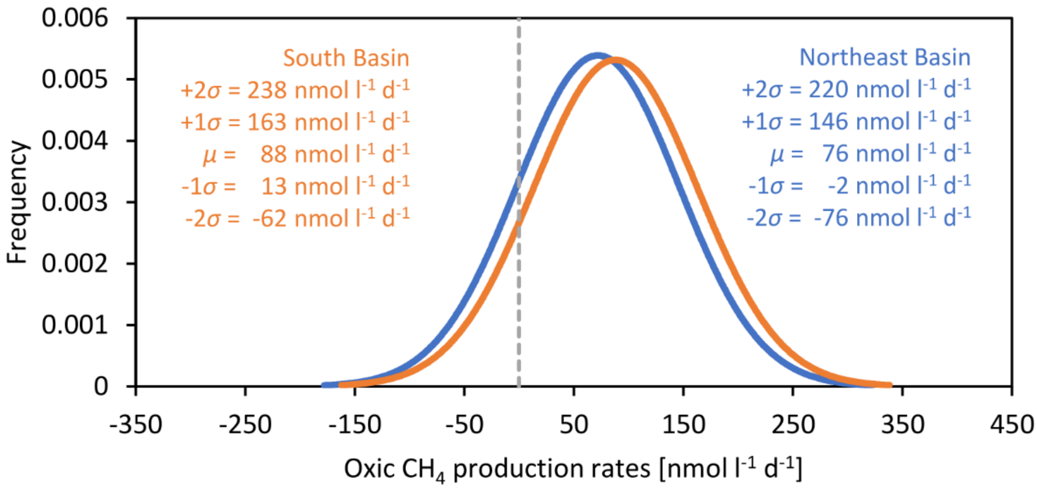

Supplementary Figure 5 | Density curve of oxic methane production rates obtained from mass balance. Monte Carlo simulation was conducted to solve methane mass balance (9999 iterations). The density function was computed as $f(x) = (1/\sqrt{2\pi}\sigma)e^{-((x-\mu)^2/(2\sigma^2))}$. σ is the standard deviation and μ is the mean value. Source data are provided as a Source Data file.

Supplementary Note 3: Estimating the oxic methane contribution (*OMC*) for additional lakes

As shown by DelSontro et al.'s[9] model for lateral transport alongside transects and actual transect measurements, methane concentrations of transect measurements reach a plateau phase at distances (corresponds to equivalent radius) ≥ 2 km (equivalent to lake surface areas > 12.6 km^2). Vertical diffusion from lower water layers into the surface mixed layer (SML) is a minor methane source, and lateral input and (oxic) internal production are the major sources of SML methane. Therefore, plateau concentrations in transect measurements represent the result of internal production while concentrations above plateau concentration represent the result of lateral transport. To estimate the dimension of both major methane sources, first the plateau concentration was integrated over the lakes' equivalent radius (resembling a measure of the oxic methane source), then elevated methane concentration were integrated over the distance of the gradient (resembling a measure of anoxic/lateral methane source) and finally the *OMC* ratio was calculated as oxic measure divided by the sum of oxic and anoxic measure. Supplementary Table 6 shows *OMC* estimations for lakes with equivalent radii above 2 km.

Supplementary Table 6 | Estimations of oxic methane contribution for lakes with equivalent radii > 2 km. Transect data were extracted from the DelSontro et al.[9] study. A graphical approach was used to compute estimates of the lateral and oxic methane sources.

Lake name	Location [coordinate North, West]	A_{tot} [km^2]	*R* [km]	z_{SML} [m]	A_{sed} [m^2]	∀ [m^3]	*OMC* [%]	*TP* [µg l^{-1}]
Beauchene (West basin)	46°39'22.7"N, 78°56'53.2"W	17	2.3	5	1.0E+05	8.5E+07	97	3.5
Champlain	44°29'07.4"N, 73°19'08.8"W	1269	20.1	10	1.3E+07	1.3E+10	100	15.2
Camichagama	47°49'54.1"N, 76°19'01.9"W	26	2.9	7	1.8E+05	1.8E+08	100	7.1
Nominingue	46°25'58.1"N, 74°59'33.0"W	22	2.7	5	1.2E+05	1.1E+08	84	8.5
Ontario	43°37'51.6"N, 77°11'06.4"W	19009	77.8	12	2.0E+07	2.3E+11	90	3.7
Simard	47°37'37.9"N, 78°43'02.3"W	170	7.3	10	6.5E+05	1.7E+09	82	21.4
St. Jean	48°31'43.0"N, 71°54'27.4"W	1065	18.4	5	8.2E+05	5.3E+09	85	9.8

A_{tot} – lake surface area, *R* – equivalent radius, z_{SML} – depth of the surface mixed layer, ∀ – volume of the surface mixed layer, *OMC* – oxic methane contribution, TP – total phosphorus level indicating the trophic state: oligo- (0 – 12 µg l^{-1}), meso- (12 – 24 µg l^{-1}) or eutrophic (>24 µg l^{-1}). Calculation of equivalent radius assumes circular shape of lake surface. *OMC* calculation assumes a maximum lateral methane transport up to 2 km.

Equivalent radius (*R*). The equivalent radius was calculated to make lakes with different morphology comparable, by assuming the lakes' surface area (A_{tot}) to be of circular shape and solving $A_{tot} = \pi R^2$ for *R*. Values for A_{tot} were derived from DelSontro et al.[9].

Volume of the surface mixed layer estimation (∀). The volume of the surface mixed layer was calculated by multiplying lake surface area (A_{tot}) with depth of the surface mixed layer (z_{SML}). Both parameters were retrieved from Supplementary Information of DelSontro et al.[9].

Sediment area estimation (A_{sed}). The sediment area was estimated by computing the perimeter of the lake from lake surface area (A_{tot}) assuming circular lake shape. The perimeter was then multiplied by the average distance (*d*) littoral sediments reach into the surface mixed layer and into depth (z_{SML}). Assuming the sediments decline at a 45°-degree angle towards lake center, *d* was computed based on the depth of the surface mixed layer (z_{SML}) and Pythagorean theorem. Morphological lake parameters needed for these transformations were derived from DelSontro et al.[9].

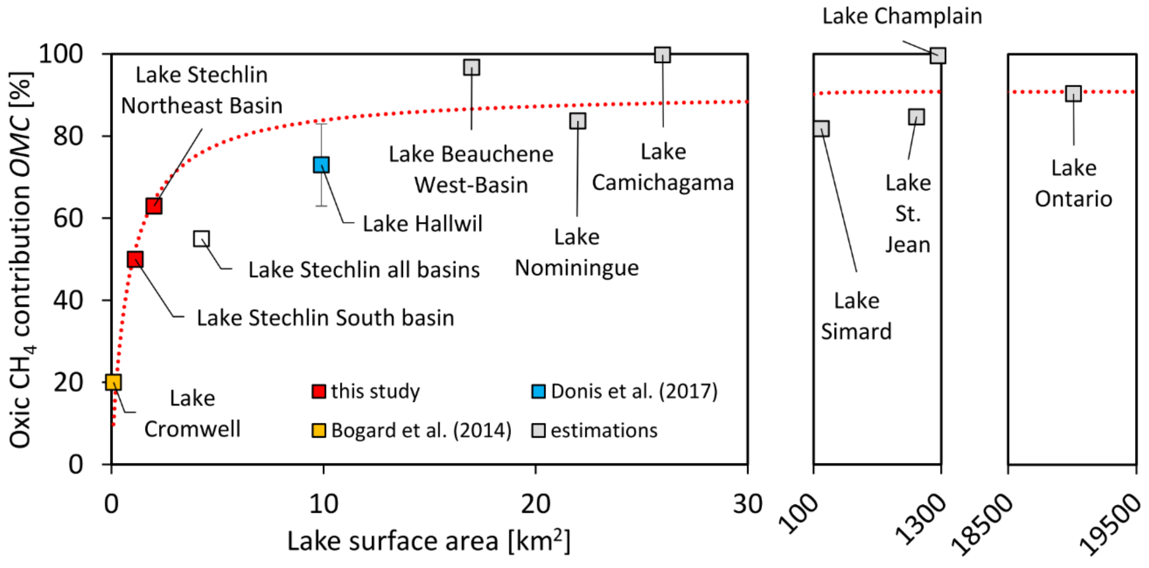

Supplementary Figure 6 | Oxic methane contribution in relation to lake size. Mass balance results, together with literature data and estimates (see Supplementary Note 3), follow a function of the type $y = (a * x)/(b + x)$. Deploying least standard error (*SE*) method gave the minimum standard error of 8.6 % when *a* = 90.87 and *b* = 0.83 (red line); indicating that the oxic source is the major SML methane source in lakes sized larger than 1 km^2. The value for Lake Hallwil was updated as described in Supplementary Note 1; the upper and lower bound estimates are represented by error bars and the mean was used to establish the trend line function. The *x*- and *y*-axes are linearly scaled. In an alternative analysis where the datasets for Lake Stechlin's two basins were combined (open square symbol), the equation constants change slightly (*a* = 92.90, *b* = 1.92, *SE* = 10.7 %). In this case the empirical model predicts the oxic methane source to be the dominant source in lakes larger than 2 km^2. Standard error *SE* was computed as $SE = \sqrt[2]{\sum(\hat{x} - x)^2/(n-2)}$ where $\hat{x}$ is the predicted value, *x* is the data point value and *n* is the number of data points. Supplementary Table 3 gives details on sampling schedules for Lake Stechlin. Source data are provided as a Source Data file.

Supplementary Table 7 | Summary of surface emission and mass balance results for Lake Stechlins' South basin using June-July 2016/2018 data and different gas transfer models.

Type of model	F_S [mmol m^{-2} d^{-1}]	P_{net} [nmol l^{-1} d^{-1}]	*OMC* [%]
Stechlin relationship	0.772±0.186	109±61	55
Hallwil relationship	0.657±0.181	73±60	45
MacIntyre et al. (2010)			
- positive buoyancy flux	0.552±0.156	41±52	32
- negative buoyancy flux	0.925±0.201	155±64	64
- combined buoyancy fluxes	0.759±0.204	104±66	54
Vachon and Prairie (2013)			
- based on wind	1.027±0.243	185±77	68
- based on wind, lake area	0.813±0.160	120±53	58

F_S – surface methane emission; P_{net} – internal (oxic) methane production; *OMC* – contribution of internal (oxic) methane production to the system-wide surface emission. Stechlin relationship was developed based on flux chamber measurements: $k_{600} = 1.98 * U_{10} + 0.94$ (k_{600} – gas transfer constant [cm h^{-1}]; U_{10} – wind speed at 10 m height [m s^{-1}]). Donis et al. (2017)[1]/Supplementary Note 1 - Hallwil relationship: $k_{600} = 2 * U_{10}$. MacIntyre et al. (2010)[4] relationships: $k_{600} = 1.74 * U_{10} - 0.15$ (at buoyancy flux $\beta > 0$); $k_{600} = 2.04 * U_{10} + 2.0$ ($\beta < 0$); $k_{600} = 2.25 * U_{10} + 0.16$ (all β). Vachon and Prairie (2013)[5] relationships: $k_{600} = 2.58 * U_{10} + 1.41$; $k_{600} = 1.48 * U_{10} + 0.39 * U_{10} * \log_{10}(LA) + 2.51$ where *LA* is lake area [km^2] (here we used basin area instead of whole-lake area). Listed values as mean±SD of 6 replicates (2016 data).

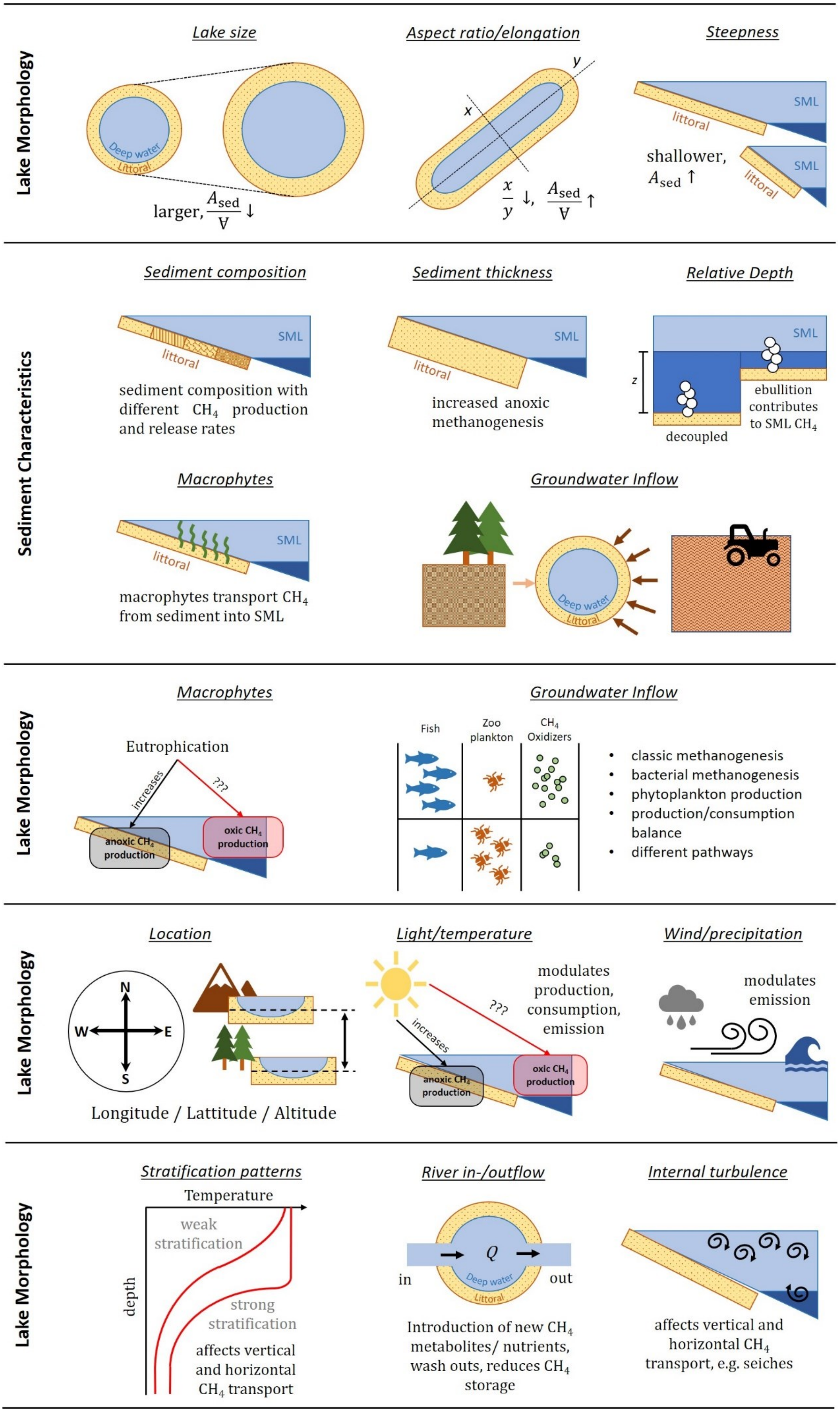

Lake Morphology
Lake size
Deep water
Littoral
larger, $\frac{A_{sed}}{\forall}$ ↓
Aspect ratio/elongation
x
y
$\frac{x}{y}$ ↓, $\frac{A_{sed}}{\forall}$ ↑
Steepness
SML
littoral
shallower, A_{sed} ↑
Sediment Characteristics
Sediment composition
sediment composition with different CH_4 production and release rates
Sediment thickness
increased anoxic methanogenesis
Relative Depth
z
decoupled
ebullition contributes to SML CH_4
Macrophytes
macrophytes transport CH_4 from sediment into SML
Groundwater Inflow
Lake Morphology
Macrophytes
Eutrophication
increases
???
anoxic CH_4 production
oxic CH_4 production
Groundwater Inflow
Fish
Zoo plankton
CH_4 Oxidizers
• classic methanogenesis
• bacterial methanogenesis
• phytoplankton production
• production/consumption balance
• different pathways
Lake Morphology
Location
N
W
E
S
Longitude / Lattitude / Altitude
Light/temperature
???
increases
modulates production, consumption, emission
anoxic CH_4 production
oxic CH_4 production
Wind/precipitation
modulates emission
Lake Morphology
Stratification patterns
Temperature
depth
weak stratification
strong stratification
affects vertical and horizontal CH_4 transport
River in-/outflow
Q
in
out
Deep water
Littoral
Introduction of new CH_4 metabolites/ nutrients, wash outs, reduces CH_4 storage
Internal turbulence
affects vertical and horizontal CH_4 transport, e.g. seiches

Supplementary Figure 7 / Examples of factors affecting the contribution of oxic and anoxic methane sources to the system-wide surface emission. Factors are categorized into lake morphology, sediment characteristics, nutrient conditions/ecology, meteorology and lake physics. A_{sed} symbolizes littoral sediment area, SML is surface mixed layer, $\forall$ refers to the volume of the surface mixed layer and Q is flow rate.

Supplementary Note 4: First-order estimation of oxic methane contribution (*OMC*) in a global context

We used the established model of *OMC* in relation with lake surface area (Supplementary Fig. 5) to get a first-order estimate of how much oxic methane production potentially contributes to the surface methane emission of lakes on a global scale. Our model is based data from temperate lakes with oligo to mesotrophic nutrient state. It is not meant to represent the biological, physical and geological complexity of all lakes; rather, it is used as a predictive tool to assess the potential global relevance of OMP based on simple lake morphology.

Global lake size class data for lakes ≥0.01 km^2 were extracted from Cael et al.[10]: this includes the abundance (*n*) and their total surface area (A_{class}; [km^2]). The percentage contribution of each lake size class to the global surface area (γ_{class}; [%]) was computed where global lake surface area (A_{global}) is 5,128,000 km^2:

$$\gamma_{\mathrm{class}} = \frac{A_{\mathrm{class}} * 100\ \%}{A_{\mathrm{global}}}\ ;\ [\%] \qquad \text{(S3)}$$

The mean size of the lake size class (A_{mean}; [km^2]), which is the average of the upper and lower limits of the size class, was applied to the *OMC*-lake size empirical model (Supplementary Fig. 6) to compute the *OMC* value for each lake size class (OMC_{class}; [%]):

$$OMC_{\mathrm{class}} = \frac{90.87 * A_{\mathrm{mean}}}{0.83 + A_{\mathrm{mean}}}\ ;\ [\%] \qquad \text{(S4)}$$

The *OMC* values for the different lake size classes (OMC_{class}) was subsequently projected to the global lake inventory relative to the total surface area (γ_{global}; [%]) by multiplying OMC_{class} values by γ_{class}:

$$\gamma_{\text{global}} = \frac{\gamma_{\text{class}}}{100\ \%} * OMC_{\text{class}};\ [\%] \tag{S5}$$

The OMC estimate for the global lake inventory (OMC_{global}; [%]) was finally computed by summing up the γ_{global} of all lake size classes:

$$\text{OMC}_{\text{global}} = \sum \gamma_{\text{global}}\ ;\ [\%] \tag{S6}$$

By accounting for the different *OMC* values of the lake size classes and the different contributions of these lake size classes to the global lake surface area, the first-order estimate for the global *OMC* was calculated to be 66 %. Supplementary Table 8 summarizes the parameters calculated for this estimation. For future applications, the model can be improved with data from different lake types, such as eutrophic lakes and lakes in other climate zones, which might deviate from the present predictive function, especially for smaller lakes.

Supplementary Table 8 I First-order estimate of the global relevance of oxic methane contribution to the surface methane emission in lakes (*OMC*). The global abundance of different lake size classes and the total surface area was extracted from Cael et al.[10] The mean lake size was applied to the established *OMC*~lake surface area model to compute *OMC* estimations for different lake size classes. These *OMC* values were then projected to the total surface area of the lake size classes.

Literature values				Applying model		Projection to global total surface area
**Lake size class [m²]**	**Abundance *n* [#]**	**Total surface area A_{class} [km²]**	**Total surface area γ_{class} [%]**	**Lake size mean A_{mean} [km²]**	**Lake size class OMC_{class} [%]**	**Global *OMC* per size class γ_{global} [%]**
$10^4 - 10^5$	23725071	683000	13.32	0.05	5.17	0.69
$10^5 - 10^6$	3813612	995000	19.40	0.5	34.21	6.64
$10^6 - 10^7$	331452	793000	15.46	5	77.96	12.06
$10^7 - 10^8$	24332	611000	11.91	50	89.39	10.65
$10^8 - 10^9$	1948	489000	9.54	500	90.72	8.65
$10^9 - 10^{10}$	211	537000	10.47	5000	90.85	9.51
$>10^{10}$	20	1020000	19.89	50000	90.86	18.07
**Σ Global**	**27896646**	**5128000**				**66.27**

Supplementary Table 9 | Mass Balance parametrization for the stratified and non-stratified periods.

Component	Terminus	Symbol	*Open Lake*		*Lake Lab Enclosures/Mesocosms (stratified condition)*	
			**Stratified condition**	**Unstratified condition**	**Enclosure E1 & E13**	**Central Enclosure**
Changing CH_4 accumulation	$\frac{\partial C}{\partial t} * \forall$		= 0 (steady state)	= 0 (steady state)	= 0 (steady state)	= 0 (steady state)
CH_4 river input	$(Q_R * C_R)$		= 0	= 0	= 0	= 0
Vertical CH_4 diffusion from metalimnion	$(A_{th} * F_z)$	A_{th} [a](NE)<br>A_{th} [a](S)<br>F_z	= 1,757,475 / 1,725,100 / 1,694,600 m^2<br>= 832,600 / 808,000 / 78,150 m^2<br>= $K_z * C'$	= 0<br>= 0<br>= $K_z * C'$	 = 63.6 m^2<br>= $K_z * C'$	 = 706.9 m^2<br>= $K_z * C'$
CH_4 sediment flux	$(A_{sed} * F_L)$	A_{sed} [a](NE)<br>A_{sed} [a](S)<br>F_L	= 249,225 / 281,600 / 312,100 m^2<br>= 290,175 / 314,775 / 335,625 m^2<br>= 1.4±0.2 mmol m^{-2} d^{-1}	= 2,006,700 m^2<br>= 1,122,775 m^2<br>= 0 mmol m^{-2} d^{-1}	 = 0 mmol m^{-2} d^{-1}<br>= 0 mmol m^{-2} d^{-1}	 = 0 mmol m^{-2} d^{-1}<br>= 0 mmol m^{-2} d^{-1}
CH_4 oxidation rate	$(MOx * \forall)$	MOx<br>$\forall$ [a](NE)<br>$\forall$ [a](S)	= 0 * P_{net}<br>= 9,439,750 / 11,197,225 / 12,922,325 m^3<br>= 4,893,575 / 5,726,175 / 6,534,175 m^3	= 0.3 * P_{net}<br>= 59,843,550 m^3<br>= 16,857,425 m^3	= 0.3 * P_{net}<br><br>= 318.1 / 381.7 / 445.3 m^3	= 0.3 * P_{net}<br><br>= 3534.3 / 4241.2 / 4948.0 m^3
Water-to-air CH_4 flux	$(A_{tot} * F_S)$	A_{tot}<br>F_S [b]	= 4,249,625 m^2<br>= measured (NE) / computed (S)	= 4,249,625 m^2<br>= measured (NE) / (S) computed	= 63.6 m^2<br>= computed	= 706.9 m^2<br>= computed
Internal CH_4 production rate	$(P_{net} * \forall)$	P_{net}<br>$\forall$ [a](NE)<br>$\forall$ [a](S)	= target parameter;<br>= 9,439,750 / 11,197,225 / 12,922,325 m^3<br>= 4,893,575 / 5,726,175 / 6,534,175 m^3	= target parameter<br>= 59,843,550 m^3<br>= 16,857,425 m^3	= target parameter<br><br>= 318.1 / 381.7 / 445.3 m^3	= target parameter<br><br>= 3534.3 / 4241.2 / 4948.0 m^3

$\forall$ - volume of the mixed layer [m^3]; ***C*** – methane concentration in the water column [mol m^{-3}]; ***t*** – time [d]; **Q_R** – inflow rate of river water [m^3 s^{-1}]; **C_R** – methane concentration in river water [mol m^{-3}]; **A_{th}** – planar area of thermocline [m^3]; **A_{sed}** – sediment area of the mixed layer; **A_{tot}** – total surface area of the lake [m^3]; **F_z** – internal turbulent diffusion from metalimnion to the surface mixed layer [mol m^{-2} d^{-1}]; **F_L** – laterally transported methane [mol m^{-2} d^{-1}]; **F_S** – water-to-air methane flux [mol m^{-2} d^{-1}]; ***MOx*** – methane oxidation rate [mol m^{-3} d^{-1}]; **P_{net}** – internal methane production [mol m^{-3} d^{-1}]; [a] depending on seasonal stage the mixed layer was either 5 (May/ June), 6 (July) or 7 m deep (August) leading to different dimensions of A_{th}, A_{sed} and $\forall$ while the whole water column was assumed to be mixed during the unstratified season; [b] methane emission at the surface was either measured using a combination of floating chamber and GC/FID unit (Northeast basin dataset; **NE**) or computed (South basin dataset; **S**) as after the linear relationship between gas transfer constants (***k***) and wind speed (U_{10}): $k \sim U_{10}$.

Supplementary Note 5: Vertical turbulent diffusivities (K_z) in the open lake and enclosures

The open-water column temperature adjacent to the mesocosm enclosures was recorded by an auto-profiler (30 min interval; between 0.5 and 20 m depth in 0.5 m increments) for the entire study period. The coefficient of the effective turbulent exchange K_{ZT} was estimated from temperature T within the water column of depth H = 20 m using the flux-gradient method[11,12]:

$$K_{zT}(z) = -\frac{\int_H^z \frac{\partial T}{\partial t}}{\left(\frac{\partial T}{\partial z}\right)_z} \text{ ; } [\mathrm{m^2\ s^{-1}}] \qquad \text{(S7)}$$

Additionally, we compared vertical diffusivities in the enclosures and in the open lake using measurements by a free-falling shear microstructure (MSS) profiler MSS-60 (ISW Wassermesstechnik) equipped with two airfoil velocity shear sensors for estimation of dissipation rate of the turbulence kinetic energy (TKE) ε, and a fast response thermistor for estimation of temperature and density fields (Prandke 2005[13]). The instrument was allowed to fall through the water column at a speed of 0.5 m s^{-1} taking measurements at 1024 Hz. To compare the vertical diffusivities inside the mesocosm enclosures and the open lake, 12 profiles were taken in one mesocosm enclosures and another 12 profiles in the open lake on 3-4 Sep 2013 during daytime at an interval of 30 min to avoid previous mixing produced by the profiler itself. The TKE dissipation rate ε was then calculated from the measured velocity shearing $\partial U/\partial z$ as described by Hinze (1959)[14]:

$$\varepsilon = \frac{15}{2}\nu\left(\frac{\partial U}{\partial z}\right)^2 \text{ ; } [\mathrm{m^2\ s^{-3}}] \qquad \text{(S8)}$$

where $\nu \approx 10^{-6}$ m^2 s^{-1} is the kinematic viscosity of water.

The mean profiles of the TKE dissipation rate were constructed by averaging over profile series in the open lake and in the enclosures and subsequently averaging over 0.25 m depth intervals. The values of ε were used to estimate the coefficient of vertical density exchange K_ρ according to Kirillin et al. (2012)[15]:

$$K_\rho = \begin{cases} C_1 \varepsilon^{1/3} z^{4/3} & at \quad \varepsilon^{1/2} N^{-3/2} \geq \kappa z \qquad \text{at the surface} \\ C_2 \varepsilon N^{-2} & at \quad \varepsilon^{1/2} N^{-3/2} < \kappa z \qquad \text{in the thermocline} \end{cases} \tag{S9}$$

where N is the buoyancy frequency in the thermocline, $C_1 = 0.4^{4/3}$ and $C_2 = 0.2$ (Osborn 1980[16]). Temperature and diffusivity profiles measured inside the mesocosms were very similar to the open-water profiles for the same period, except the upper several meters of the epilimnion, where wind mixing produced a stronger turbulence in the open lake (Supplementary Fig. 8). This allowed us to apply the same heat-budget estimates of open-water diffusivity values at depths >4 m to estimate the vertical flux in both open lake and mesocosm enclosures for the entire study period.

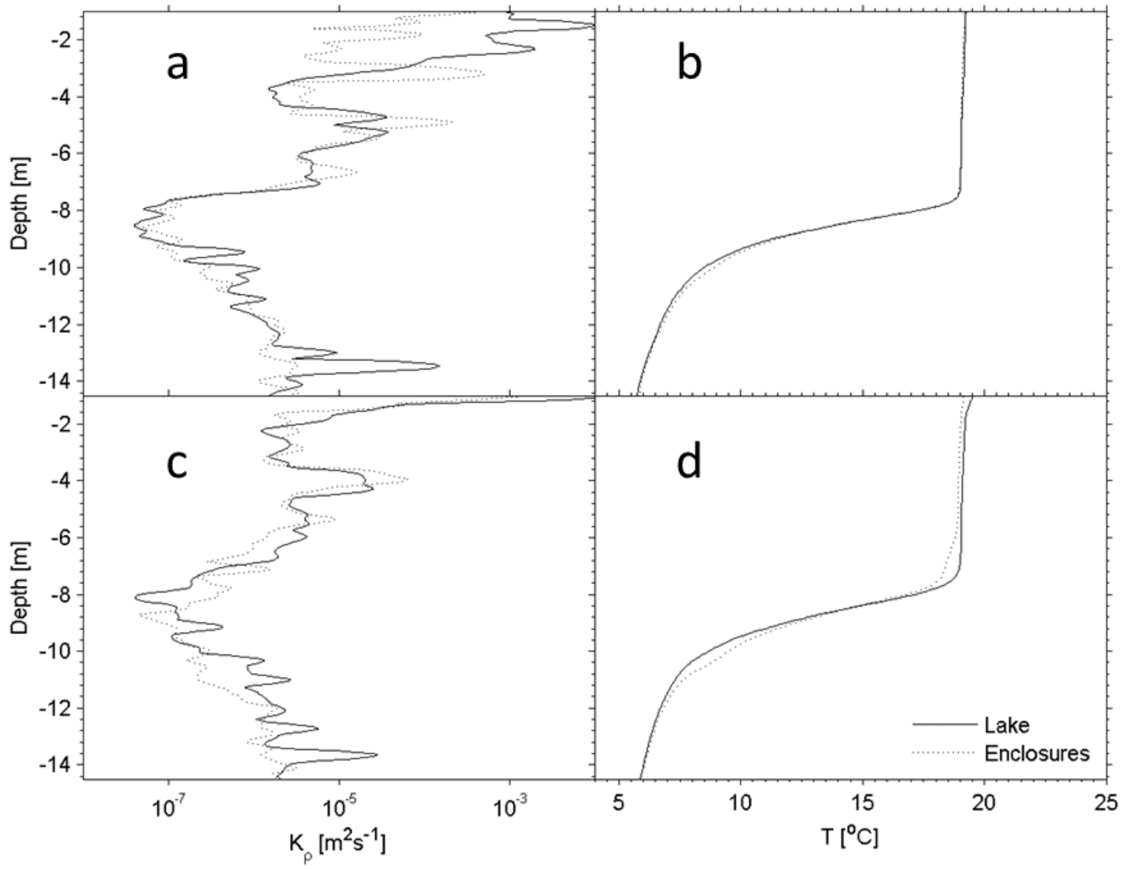

Supplementary Figure 8 | Physical parameters inside enclosures and in the open lake. Vertical turbulent diffusion coefficient (**a**, **c**) and vertical temperature distribution from the MSS profiling (**b**, **d**) in the mesocosm enclosures (dotted lines) and in the open lake (solid lines) under windy condition (Panels **a**-**b**, 3 Sep 2013, wind speeds 3-5 $m\,s^{-1}$) and calm condition (Panels **c**-**d**, 4 Sep 2013). Source data are provided as a Source Data file.

Supplementary References

1. Donis, D. et al. Full-scale evaluation of methane production under oxic conditions in a mesotrophic lake. *Nat. Commun.* **8,** 1661 (2017).
2. Bierlein, K. A. et al. Increased sediment oxygen flux in lakes and reservoirs: the impact of hypolimnetic oxygenation. *Water Resour. Res.* **53,** 4876-4890 (2017).
3. Flury, S., et al. Methane emissions from a freshwater marsh in response to experimentally simulated global warming and nitrogen enrichment. *J. Geophys. Res.* **115,** G01007 (2010).
4. MacIntyre, S. et al. Buoyancy flux, turbulence, and the gas transfer coefficient in a stratified lake. *Geophys. Res. Lett.* **37,** L24604 (2010).
5. Vachon, D. & Prairie, Y. T. The ecosystem size and shape dependence of gas transfer velocity versus wind speed relationships in lakes. *Can. J. Fish. Aquat. Sci.* **70,** 1757-1764 (2013).
6. Winslow, L. et al. rLakeAnalyzer: Lake Physics Tools. R package version 1.11.4 at https://CRAN.R-project.org/package=rLakeAnalyzer (2018).
7. Engle, D. & M. Melack, J. Methane emissions from an Amazon floodplain lake: enhanced release during episodic mixing and during falling water. *Biogeochemistry* **51,** 71–90 (2000).
8. Jähne, B. J. et al. On the parameters influencing air-water gas exchange. *J. Geophys. Res.* **92,** 1937–1949 (1987).
9. DelSontro, T., del Giorgio, P. A. & Prairie, Y. T. No longer a paradox: the interaction between physical transport and biological processes explains the spatial distribution of surface water methane within and across lakes. *Ecosystems* **21,** 1073-1087 (2018).

10. Cael, B. B., Heathcote, A. J. & Seekell, D. A. The volume and mean depth of Earth's lakes. *Geophys. Res. Lett.* **44,** 209–218 (2017).

11. Powell, T. & Jassby, A. The estimation of vertical eddy diffusivities below the thermocline in lakes. *Water Resour. Res.* **10,** 191-198 (1974).

12. Dubovskaya, O. P. et al. Effects of water column processes on the use of sediment traps to measure zooplankton non-predatory mortality: a mathematical and empirical assessment. *J. Plankton Res.* **40,** 91-106 (2017).

13. Prandke, H. in Marine Turbulence: Theories, Observations, and Models (eds Baumert, H., Simpson, J. & Sündermann, J.) 101-109 (Cambridge Univ. Press, Cambridge, 2005).

14. Hinze, J. O. *Turbulence* (McGraw-Hill, New York, 1959).

15. Kirillin, G., Grossart, H.-P. & Tang, K. W. Modeling sinking rate of zooplankton carcasses: effects of stratification and mixing. *Limnol. Oceanogr.* **57,** 881-894 (2012).

16. Osborn, T. R. Estimates of the local rate of vertical diffusion from dissipation measurements. *J. Phys. Oceanogr.* **10,** 83–89 (1980).
