# Supplementary material for: Contribution of oxic methane production to surface methane emission in lakes and its global importance
Source: Nat Commun. 2019 Dec 2;10:5497. doi: 10.1038/s41467-019-13320-0 (PMC6888895; doi:10.1038/s41467-019-13320-0)
Supplement: Supplementary file 3 — Reporting Summary [file 41467_2019_13320_MOESM3_ESM.pdf]

nature research

Corresponding author(s): Marco Günthel

Last updated by author(s): 2019-10-29

Reporting Summary

Nature Research wishes to improve the reproducibility of the work that we publish. This form provides structure for consistency and transparency in reporting. For further information on Nature Research policies, see Authors & Referees and the Editorial Policy Checklist.

Statistics

For all statistical analyses, confirm that the following items are present in the figure legend, table legend, main text, or Methods section.

n/a	Confirmed	
☐	☒	The exact sample size (*n*) for each experimental group/condition, given as a discrete number and unit of measurement
☐	☒	A statement on whether measurements were taken from distinct samples or whether the same sample was measured repeatedly
☐	☒	The statistical test(s) used AND whether they are one- or two-sided<br>*Only common tests should be described solely by name; describe more complex techniques in the Methods section.*
☒	☐	A description of all covariates tested
☐	☒	A description of any assumptions or corrections, such as tests of normality and adjustment for multiple comparisons
☐	☒	A full description of the statistical parameters including central tendency (e.g. means) or other basic estimates (e.g. regression coefficient) AND variation (e.g. standard deviation) or associated estimates of uncertainty (e.g. confidence intervals)
☐	☒	For null hypothesis testing, the test statistic (e.g. *F*, *t*, *r*) with confidence intervals, effect sizes, degrees of freedom and *P* value noted<br>*Give P values as exact values whenever suitable.*
☐	☒	For Bayesian analysis, information on the choice of priors and Markov chain Monte Carlo settings
☒	☐	For hierarchical and complex designs, identification of the appropriate level for tests and full reporting of outcomes
☐	☒	Estimates of effect sizes (e.g. Cohen's *d*, Pearson's *r*), indicating how they were calculated

Our web collection on statistics for biologists contains articles on many of the points above.

Software and code

Policy information about availability of computer code

Data collection: not applicable

Data analysis: not applicable

For manuscripts utilizing custom algorithms or software that are central to the research but not yet described in published literature, software must be made available to editors/reviewers. We strongly encourage code deposition in a community repository (e.g. GitHub). See the Nature Research guidelines for submitting code & software for further information.

Data

Policy information about availability of data

All manuscripts must include a data availability statement. This statement should provide the following information, where applicable:

- Accession codes, unique identifiers, or web links for publicly available datasets
- A list of figures that have associated raw data
- A description of any restrictions on data availability

Data are made available in graphical or tabular form throughout the manuscript and Supplementary Information. The source data underlaying Figs. 2, 3 and 4 and Supplementary Figures 1, 3, 4, 5, 6 and 8 are provided as a Source Data file.

Field-specific reporting

Please select the one below that is the best fit for your research. If you are not sure, read the appropriate sections before making your selection.

☐ Life sciences ☐ Behavioural & social sciences ☒ Ecological, evolutionary & environmental sciences

For a reference copy of the document with all sections, see nature.com/documents/nr-reporting-summary-flat.pdf

Ecological, evolutionary & environmental sciences study design

All studies must disclose on these points even when the disclosure is negative.

Study description	The system-wide methane sources and sinks in the surface mixed layer of the mid-water column has been balanced in two basins of Lake Stechlin. In addition, the mass balance has been performed for 2 experimental enclosures (subject to periodic water exchange) and the central reservoir (no water exchange for many years). The enclosures/the reservoir are cut off from lateral methane input. The mass balances revealed that a substantial quantity of methane source is missing when only anoxic methane sources (+ sinks) are considered. The contribution of the missing mass balance component (internal oxic methane production) to surface emission was computed for both basins separately (and combined for whole-lake consideration). Data collected for during the mixed and stratified season were considered separately (each basin). Monte Carlo Simulation was applied to obtain average values. This results were finally combined with literature data to evaluate the general trend of the contribution pattern of the oxic methane source to surface emission (regression analysis of a data set with n=11 or n=10 respectively).
Research sample	The following water columns have been sampled:<br>experimental enclosure 1 in the South basin (n=4, stratified season); experimental enclosure 13 (n=5, stratified season); open water of the South basin (n=7, stratified season); open water of the South basin (n=10, mixed season); open water of the Northeast basin (n=7, stratified season); open water of the Northeast basin (n=13, mixed season); central reservoir of the South basin (n=1, stratified season); central reservoir of the South basin (n=2, mixed season). Note, each replicate includes 2 or 3 methodological replicates.
Sampling strategy	Samples of the open lake which were used for downstream analysis and retrieving the overall conclusion were taken weekly from March to July 2016 with an additional repitition in July 2018. The sample sized of n=14 (South and Northeast basin combined) substantially increases the data resolution per lake compared to earlier studies published in Nature Communications, i.e. Bogard et al. 2014 (doi.org/10.1038/ncomms6350), Donis et al. 2017 (doi.org/10.1038/s41467-017-01648-4). Note, to ensure the conclusion retrieved from our dataset is correct, we performed (1) a sensitivity analysis deploying a variety of alternative and very conservative mass balance parametrizations, and (2) we used Monte Carlo Simulation to obtain mean values (deduced from average values of field measurements and their standard deviation).
Data collection	Marco Günthel collected the field data together with Hans-Peter Grossart, Danny Ionescu and Mina Bizic. Water samples were taken from boat (Northeast basin) or the Lake Lab platform (South basin, enclosures, central reservoir) using a Limnos Water sampler, transferred to serum bottles, 3 times flushed and crimp-closed without bubble enclosure (1or 2 m depth resolution; 2 to 3 methodological replicates each). Methane concentration was finally recorded after headspace displacement technique and GC/FID analysis (2 additional methodological replicates). Surface methane emission was quantified using 3 approaches: (1) using a 15 l-flux chamber (concurrent to the concentration sampling; increasing methane content in the flux chamber was measured over time; as syringe was used to collect trapped gas aliquots which were transferred into 50 ml serum bottles prefilled with NaCl saturated double distilled water, concentration was retrieved by headspace displacement technique and GC/FID analysis; the final surface flux was retrieved by timely regression of the methane content and relating to the the chamber profile area; 9 time points for regression per value); (2) surface emission was quantified by k600-wind relationship developed from own surface emission and wind data (10 m height) provided from the Umweltbundesamt (weatherstation adjacent to the lake) in 30-60 min intervals. Oxygen and temperature was recorded using automatic probes recording the temperature in a 30 min interval with a 0.5 m depth resolution.
Timing and spatial scale	Data was collected during daytime (10:00-18:00 local time) in 2016 and 2018: experimental enclosure 1 in the South basin (n=4, stratified season, Aug 2014); experimental enclosure 13 (n=5, stratified season, , Aug 2014); open water of the South basin (n=7, stratified season, Jun/Jul 2016); open water of the South basin (n=10, mixed season, Mar-May); open water of the Northeast basin (n=7, stratified season, Jun/Jul 2016); open water of the Northeast basin (n=13, mixed season, Mar-May); central reservoir of the South basin (n=1, stratified season, Jul 2016); central reservoir of the South basin (n=2, mixed season, May).
Data exclusions	No data has been excluded.
Reproducibility	Measurements have taken over a seasonal change with in a high-resolution time interval of approximately weekly sampling from Mar 2016 to July 2016 in 2 different lake basins. Each system has been sampled for n=4-13 times.
Randomization	Sampling of open water sampling sites were done at the deepest point of the lake (Northeast basin) and adjacent to the Lake Lab facility (South basin). The deepest point resembles the water column with the least influence of lateral methane input whereas adjacent to the lake a strong lateral influence is expected (proximity to shore and littoral sediments). The sampling schedule (approx. weekly) was dependent on boat availability and was not made dependent on weather condition. Note, 4 different people handled measurements of field parameters and their individual contributions to the sampling schedule were randomly distributed over time.
Blinding	This study is based on field measurements and did not include experiments associated to different treatment groups (i.e. bottle incubation experiments). However, 4 different peoples (random schedules) contributed to field measurements.

Did the study involve field work? ☒ Yes ☐ No

Field work, collection and transport

Field conditions	The sampling schedule was independent of field conditions. This study is based on field measurements taken over the seasonal change from Mar to Aug (combining 2014, 2016, 2018) with a weekly sampling schedule in 2016 from Mar-Jul. Accordingly, a variety of weather conditions prevailed during the study period: surface water temperatures of ca. 3-24°C, wind speed of 0-9 m/s, occasional mild rain. Details are laid out in the manuscript and supplementary materials.
Location	open water South basin (53°08'36.6"N 13°01'42.8"E, ca. 20.5 m deep),<br>open water Northeast basin (53°09'20.2"N 13°01'51.5"E, ca. 69.5 m deep),

	experimental enclosure 1 (53°08'36.4"N 13°01'41.6"E; ca. 20 m deep),<br>experimental enclosure 13 (53°08'36.5"N 13°01'42.1"E; ca. 20 m deep),<br>central reservoir (53°08'35.8"N 13°01'41.1"E; ca. 18.5 m deep);<br>note, the surface mixed layer was 5 to 7 m deep depending on the time of the year
Access and import/export	Sampling in Lake Stechlin required boat usage and has been done in agreement with and under supervision by the Leibniz Institute of Freshwater Ecology and Inland Fisheries (IGB Berlin; adjacent to the lake) following the local safety protocols. The IGB institute monitors Lake Stechlin for decades and is permitted to use boats in Lake Stechlin.
Disturbance	The motor engine of boats can artificially disturb the methane content inside the water column and affect the emission. Therefore, to avoid this disturbance, the boat was attached not at the final sampling side but at a different site of the Lake Lab facility (South basin) or attached to a swimming platform over the deepest point and allowed to float freely as modulated by the wind (Northeast basin) away from the disturbed water column.

Reporting for specific materials, systems and methods

We require information from authors about some types of materials, experimental systems and methods used in many studies. Here, indicate whether each material, system or method listed is relevant to your study. If you are not sure if a list item applies to your research, read the appropriate section before selecting a response.

Materials & experimental systems

n/a	Involved in the study
☒	☐ Antibodies
☒	☐ Eukaryotic cell lines
☒	☐ Palaeontology
☒	☐ Animals and other organisms
☒	☐ Human research participants
☒	☐ Clinical data

Methods

n/a	Involved in the study
☒	☐ ChIP-seq
☒	☐ Flow cytometry
☒	☐ MRI-based neuroimaging
